# Special Hospitals

**Published:** 1861-04

**Authors:** 


### Art. X.—SPECIAL HOSPITALS.

The active war declared last year by the supporters of General Hospitals against the further spread of Specialism and Special Hospitals, seems to have degenerated into a chronic scuffle, mainly kept alive by the committee appointed to inquire into the question by the last General Meeting of the British Medical Association. Of the long array of names that followed the leading of Sir B. Brodie in the famous protest, not one seems inclined to pursue the matter further. We are not surprised at this, inasmuch as professional men are always found to be wonderfully gregarious, under the leadership of great names, in opposing innovations of any kind. They will fight under the standard of authority; but, in such combinations of men, not many will be found to step out of the ranks in order to do battle on their own account. Probably the Sir B. Brodie protest has since been discovered to leak most wofully. A very large number of those who signed it, and thus thought to wash their hands of the unclean thing Specialism, have been discovered to possess a weakness towards pet specialities of their own, but which, of course, they think as veritable exceptions to the general rule, as ophthalmic hospitals, so specially and unaccountably withdrawn from the general censure by our great chief himself. In short, there appears to have been a very prevalent idea among them that theirs are the only doxys free from heterodoxy. Standing, as we do, between the combatants, and having no special relations with either, we may be allowed to examine the matter philosophically. Is this Specialism a natural growth of our civilization, or is it merely the offspring of self-seeking? We cannot help thinking that the springs of the movement are to be sought in both directions. If Specialism is an unadulterated evil, we may be permitted to ask how the long recognised distinction between physician and surgeon ever came to be permitted? Why are we not all general practitioners, as they are in country districts? The answer is clear enough. The profession simply adapts itself to the conditions in which it is placed: in the country, a medical man must be able to turn his hand to every want of the profession if he would live. In the ordinary run of cases, the general practitioner is quite up to his work; but in all cases of special difficulty, he is the first himself to seek the advice of the special man in the neighbouring city. In lesions of the chest, he sends for Dr. J. C. Williams; for a difficulty in the eye, Dr. Beaumont is summoned; for a critical operation, Mr. Ferguson's services are required, and so on. The general practitioner, every day of his life, pays this compliment to Specialism. It cannot, therefore, with any justice, be urged that Specialism is *per se* indefensible.

Robinson Crusoe was obliged to turn a cabinet-maker, agriculturist, boat-builder, stock-breeder, &c.; but when he was rescued from his island, he would most indubitably have bought a suit of broadcloth of the tailor instead of trying his hand again at goatskins. We cannot ignore the fact that, in all professions or trades, division of labour is being carried to an astounding extent. In the army, we find the three arms of the service having duties, and holding interests totally distinct from each other. The profession of law branches off into special pleaders, conveyancers, equity draughtsmen, and many other shades of forensic Specialism. In the engineering ranks how diverse are the paths trodden by gentlemen all originally educated for the same general profession. It is simply absurd to say that medicine is to be an exception to this general and very necessary tendency. The study of the diseases of the human body is a much wider one than that of either law or arms: the human intellect is not capable of minutely examining every detail of this inexhaustible subject, and, therefore, if we would really be proficient, and follow nature with subtlety and success, we must limit the field of our vision. Granted, replies the advocate of Generalism; but this is a question of degree, Specialism has gone far enough. When the chimney-sweep in *Pickwick* demands to be shaved, the barber magnificently waves him off with the reply, "We must draw the line somewhere—we stops at bakers!" Sir Benjamin Brodie draws the line at ophthalmic hospitals; but it is scarcely necessary to state, Specialism will decline to be shaved so far and no farther, even by the authority of the greatest name. It may suit the purpose of those practising Specialities, made orthodox by custom, to pooh pooh the introduction of new ones, and to denominate them as innovations, but they cannot do so with any shadow of reason, and they know they cannot. In speaking of Specialism, we have of course taken it for granted, that if it is good, Special Hospitals must also be good. The reason why a Specialist is a better practitioner in his particular line than a General practitioner, is simply because he has greater experience than the latter. He sees a thousand cases where the other sees only ten; consequently his powers of discrimination are enhanced, and he is far more likely to get at the general law of the case than the practitioner who is obliged to diagnose from cases few and far between. The congregation of like cases in a hospital gives an immense teaching power to the lecturer, which students know well enough. If a man wishes to study orthopædic surgery, he does not go to St. Bartholomew's, or Guy's, but to the Orthopædic Hospital; again, if wishing to work at the eye, he prefers the Ophthalmic Hospital to King's College. These Special Hospitals are equally instructive to teacher and pupil: to the former it affords manipulative dexterity (sharpened to the highest point by being directed upon a limited object), as well as diagnostic power of a very high order; to the latter, the fullest possible view of the subject under study in every aspect. And can it be denied that medicine itself will be a great gainer by this division of labour? It is admitted on all hands that what has been wanted towards evolving trustworthy laws of disease, is a comprehensive and exact system of medical statistics. At present we dogmatize upon a few hundred cases, and those not viewed side by side as they would be in a Special Hospital, but picked out of the confused heap of cases that are passed in review in a General Hospital, these cases extending over so long a period of time that minute accuracy of comparison between their different distinctive features is quite out of the question. We have no hesitation in saying that medical statistics, or the "numerical method," as Dr. Guy terms it in his admirable lecture, will never be built up from the records of our General Hospitals as at present conducted, and we are equally certain that Special Hospitals will afford us this new aid towards a more correct view of disease. Is it reasonable, we ask, that classification should be adopted in every other scientific inquiry, but denied to medicine?

But, says the advocate for the established order of things, if our great hospitals are to be broken up into petty institutions, what is to become of our schools?—how will all the students be able to find time or money to go the round of a score of institutions for the treatment of all the ills that flesh is heir to? This is a very pertinent question, and one requiring a satisfactory answer. We confess we do not wish to see the study of disease, which should be one grand unity, frittered away in detail. We have no desire to find the human economy treated like the mechanism of a watch, in the study of which one man confines himself to wheels, another to balance springs, and a third to mainsprings, whilst each artisan is ignorant of the handiwork of his neighbour. If Special Hospitals lead to this result, we would say, Away with them; if the teachers in them could only give sketches of disease, which the student would have afterwards to put together with more than the neatness of a Chinese puzzle, if the effect of one disease upon another were thus lost to his comprehension, we should cast in our vote with the General Hospital memorialists. But Specialism has never contemplated such a state of things. Every Specialist receives his general education before he branches off to some limited line of practice.

In the Royal Academy the student must first become versed in general principles, and attain accuracy of eye and hand. These preliminaries mastered, he is free to take up miniature, landscape, or historical painting. If the teachers in our great schools cannot, under existing circumstances, ground the student in the general principles of medicine so as to afford a firm foundation from which he may direct his studies in any special direction, the fault is theirs. It is urged, we know, that the spread of Special Hospitals drains the General Hospital of its best cases, and thus reduces the teaching power of our great schools. That there is a tendency in this direction we most readily admit, but we must also observe that the inefficiency of the General Hospitals has brought on this state of things. Why have their supporters not seen that classification of disease must take place sooner or later either inside or outside of their walls? With the example of the magnificent hospitals of Vienna before their eyes, in which we find special diseases treated in special wards, why have they allowed "outsiders" to take up the running, and to establish these special wards as Special Hospitals? If we examine the plan on which all modern Lunatic Asylums are built, we find classification carried to the utmost limit. Why should classification, found to be so useful in mental diseases, not be tried also in bodily diseases? The answer is, that asylums are generally of modern construction and under Government control, whilst hospitals are in too many cases under a system of government which desires no change, no departure from the old routine, which manufactures medical officers on the same principle that machine-made bricks are now constructed—the one behind pushing the one before forward, and forcing him out a ready-made brick (or officer) exactly according to pattern. The consequence of this system of rotation, a thousand times more inexorable than that obtaining in the army, is that good and true men, who have proved their quality, if "unattached," can find no place, and are forced to establish a field for their exertions in

Special Hospitals. Can we wonder that Brown-Séquard has thus been forced to found a hospital for epileptics? Is it a wonder that Marshall Hall contemplated a similar institution, in consequence of having no hospital appointment in which he could exercise his undoubted genius? We think, then, we have traced the establishment and rapid spread of Special Hospitals to two sources: 1, The spread of civilization, which inevitably leads to classification and the division of labour; 2, The inefficiency and the cliqueism of the General Hospitals. These two causes, operating in the same direction, have brought on a revolution which conservative medicine beholds in full career, and vainly seeks to stop with an empty and hypocritical protest.

